# DNA barcodes and evidence of cryptic diversity of anthropophagous mosquitoes in Quintana Roo, Mexico

**DOI:** 10.1002/ece3.5073

**Published:** 2019-03-23

**Authors:** Rahuel J. Chan‐Chable, Arely Martínez‐Arce, Pedro C. Mis‐Avila, Aldo I. Ortega‐Morales

**Affiliations:** ^1^ Departamento de Sistemática y Ecología Acuática Unidad Chetumal, El Colegio de la Frontera Sur Chetumal Quintana Roo México; ^2^ Departamento de Enfermedades Transmitidas por Vector y Zoonosis Servicios Estatales de Salud de Quintana Roo Chetumal Quintana Roo México; ^3^ Universidad Autónoma Agraria Antonio Narro Torreón Coahuila México

**Keywords:** anthropophagous, cryptic diversity, DNA barcodes, Mexico, mosquitoes

## Abstract

Culicidae mosquitoes are potential vectors of pathogens that affect human health. The correct species identification, as well as the discovery and description of cryptic species, is important in public health for the control and management of specific vectors. In the present study, the diversity of anthropophagous mosquitoes in Quintana Roo, at the border between Mexico and Belize, was evaluated using morphological and molecular data (COI‐DNA Barcoding). A total of 1,413 adult female specimens were collected, belonging to eight genera and 31 morphospecies. Most species formed well‐supported clades. Intraspecific Kimura 2 parameters (K2P) distance average was 0.75%, and a maximum distance of 4.40% was observed for *Anopheles crucians*s.l. ABGD method identified 28 entities, while 32 entities were identified with the BIN system. In *Culex interrogator* and *Culex nigripalpus* a low interspecific genetic distance of 0.1% was observed. One undescribed species belonging to the genus *Aedes* (*Aedes*n. sp.) was discovered, but no clear genetic divergence was found between this species and the closely related species *Aedes angustivittatus*. An intraspecific K2P distance greater than 2.7% was observed in *Aedes serratus*(3.9%), *Anopheles crucians*s.l. (4.4%), *Culex taeniopus* (3.7%), *Haemagogus equinus* (3.9%), *Culex erraticus* (5.0%), *Psorophora ferox* (4.5%), and in *Anopheles apicimacula*(8.10%); therefore, evidences of cryptic diversity are shown in these species. This study showed that DNA barcodes offer a reliable framework for mosquito species identification in Quintana Roo, except for some closely related species for which it is recommended to use additional nuclear genetic markers such as ITS2, in order to resolve these small discrepancies.

## 1 INTRODUCTION

Recent studies indicate that it is necessary to keep identifying mosquito species in different regions of the world through the use of traditional taxonomy and DNA sequences, to achieve a better estimation of mosquito biodiversity (Ashfaq et al., 2014; Azari‐Hamidian et al., 2010; Batovska, Blacket, Brown, & Lynch, 2016; Cywinska, Hunter, & Hebert, 2006; Rozo‐López & Mengual, 2015; Versteirt et al., 2015; Wang et al., 2012; Weeraratne, Surendran, & Parakrama Karunaratne, 2018). The combined use of DNA and morphology allowed to analyze a greater number of species worldwide and to discover cryptic speciation in taxa of medical importance such as culicides (Ashfaq et al., 2014; Hebert & Gregory, 2005; Laurito, Oliveira, Almirón, & Sallum, 2013; Torres‐Gutiérrez et al., 2016; Wang et al., 2012). Though, given the diversity of mosquitoes, it is possible to affirm that integrative taxonomy in this group is at its early stages (Beebe, 2018).

For several years, the Cytochrome Oxidase I subunit (COI) gene as a molecular marker for mosquitoes, has evidenced the presence of species complexes and cryptic species within the genera *Aedes*, *Anopheles* and *Culex* (Ashfaq et al., 2014; Wang et al., 2012). These discoveries have been interesting from an ecological point of view, as molecular analyses demonstrated a wide distribution of several genera, and predicted that speciation processes may be occurring at an unobservable rate due to the constant exposure to physical and biological environmental factors. Therefore, molecular information in addition to predicting the presence of a great number of undescribed species also allows analyzing the variation at the genetic level (Weeraratne et al., 2018).

The discovery of cryptic species in Culicidae also entails serious public health implications as it directly impacts the design of vector control and management programs (Bickford et al., 2007). The correct identification of the species is essential for the development of programs for the control and prevention of diseases transmitted by mosquitoes, as it allows focusing only on the control of those species that transmit certain diseases (Azari‐Hamidian et al., 2010; Bueno‐Marí, Corella‐López, & Jiménez‐Peydró, 2010; Erlank, Koekemoer, & Coetzee, 2018; Laboudi et al., 2011).

Unlike other groups of insects, Culicidae mosquitoes are widely studied due to their role in human health (Cardoso et al., 2010; Rozo‐López & Mengual, 2015). Nevertheless, their knowledge from a taxonomic point of view is still uncomplete (Kumar, Rajavel, Natarajan, & Jambulingam, 2007). The difficulties in the correct identification of mosquito species derive from the impossibility to use some diagnostic characters, such as scales and setae, which get often damaged during field collection or storage of specimens, and the presence of other characters which are evident only during some stages of their growth (Kumar et al., 2007). Furthermore, isomorphic species can be often found in mosquitos, grouped in closely related species (sibling species) (Beebe, 2018). These issues cause delays in the correct identification of the species (Ruiz‐López et al., 2012), besides the fact that a high taxonomic expertise in needed.

There are 3,554 species of mosquitoes recognized worldwide (Harbach, 2018), of these only 1,254 have been barcoded that is <40% (www.Boldsystems.org) (Ratnasingham & Hebert, 2007). In Mexico, around 250 species belonging to 20 genera are reported (Bond et al., 2014; Ibáñez‐Bernal, Strickman, & Martínez‐Campos, 1996; Ortega‐Morales et al., 2015). For Quintana Roo there of 79 species in 15 genera are recorded (Chan‐Chable, Ortega‐Morales, & Martínez‐Arce, 2016; Ordóñez‐Sánchez et al., 2013; Ortega‐Morales et al., 2015; Salomón‐Grajales et al., 2012). Currently, most of the nominal species in the region have been identified based on traditional taxonomy and only about 45 species have been barcoded (R. J. Chan‐Chable & A. Martínez‐Arce, Unpublished data).

Among the important genera reported for Quintana Roo is the genus *Aedes*sensu Wilkerson et al. (2015) potential vector of pathogens causing diseases such as yellow fever, dengue, zika, among other diseases that represent a threat to human and animal health (Ortega Morales et al., 2010; Salomón‐Grajales et al., 2012). In the present study, a total of nine species of *Aedes* genus were identified, although the number of species belonging to this genus is present in the State is still unknown if exist more due to the lack of exhaustive studies involving morphology and DNA barcoding, for this and other species of medical importance.

The objectives of this study were to delimit anthropophagous mosquito species using DNA barcodes and traditional taxonomy in the southeastern region of Mexico, an area designated as malarial on the border between southern Quintana Roo and Belize; to find evidences of cryptic species in designated species with wide distribution and, finally, to contribute to GenBank and Bold systems databases with new DNA barcodes.

## 2 MATERIAL AND METHODS

### 2.1 Mosquitoes collection

**Figure 1:**
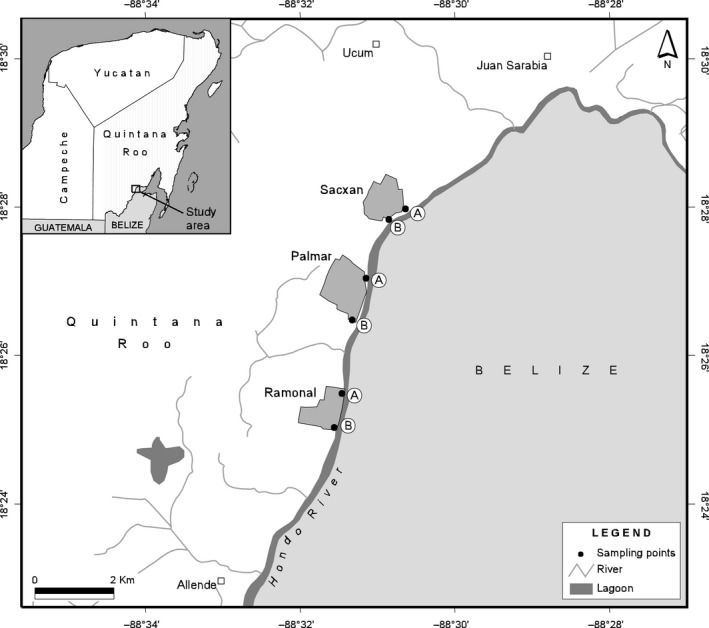
Study area. The three sampling locations are shown, A and B are the sampling sites by location

Adult females were collected in three selected locations of Quintana Roo, Mexico (Sacxan, Palmar, and Ramonal) on the border between Mexico and Belize (two sites per location: Sacxan (A): 18°27′52.76″N–88°30′58.44″W, (B): 18°27′44.61″N–88°31′08.44″W; Palmar (A): 18°26′43.0″N–88°31′27.4″W, (B): 18°26′26.9″W–88°31′37.99″W; Ramonal (A): 18°25′27.7″N–88°31′45.97″W, (B): 18°25′08.1″N–88°31′47.7″W (Figure 1). The collection sites were an altitude of 10–20 m and currently are cataloged as endemic areas of dengue and malaria transmission (SINAVE, 2018). Mosquito collections were conducted in each location from 6:00 to 9:00 and from 18:00 to 21:00 hr during September–December 2015 with the help of the Mexican public health official personnel, taking preventive measures for the collection of Anophelines from southern Mexico and Central America (Méndez‐Galván, Betanzos‐Reyes, Velázquez‐Monroy, & Tapia‐Conyer, 2004). Mosquitoes were collected using aspirator tubes while they approached to the collecting personnel. All specimens collected were sacrificed in glass jars with ethyl acetate vapors and transported to the Zoology Laboratory of ECOSUR‐Chetumal Unit where they were separated by species, mounted on entomological pins and photographed.

### 2.2 Depository and morphological identification

Vouchers were deposited in the Entomology Collection of the Zoology Museum of ECOSUR‐Chetumal Unit where they were assigned a reference code (ECO‐CH‐AR/DP_0285‐1697), and lateral view photographs of each voucher were taken. All specimens were identified using different available literature such as books and identification keys, which included Díaz Nájera (1965), Berlin (1969), Arnell (1976), Darsie and Ward (1981), Sirivanakarn (1982), Clark‐Gil and Darsie (1983), and Wilkerson and Strickman (1990). The collected data were deposited in the BOLD Systems^®^ database (www.boldsystems.org) (Ratnasingham & Hebert, 2007) under the project “Mosquitoes from Rio Hondo, Mexican Caribbean” (MRRO). The Culicidae classification system proposed by Wilkerson et al. (2015) and available in the Walter Reed Biosystematic Unit (www.wrbu.org) was used for this study.

### 2.3 DNA isolation, PCR amplification, and sequencing

For the molecular analysis, a total of 272 specimens that represented 31 morphospecies, were selected. Five to thirty specimens per morphospecies were selected. All specimens were processed by removing one hind leg from each individual, which was deposited in a sterile tube of 0.2 ml with 30 µl of ethanol (Ashfaq et al., 2014). DNA extraction and PCR amplification were performed following the protocols of Ivanova, deWaard, and Hebert (2006). For the amplification of COI–5′ gene, two sets of primers were used LCO1490/HCO2198 and LCO1490_t1/HCO2198_t1 (Folmer, Black, Hoeh, Lutz, & Vrijenhoek, 1994; Foottit, Maw, Havill, Ahern, & Montgomery, 2009). The following thermocycling conditions were used: initial denaturation of 1 min at 94°C, followed by five cycles of 94°C for 40 s, 45°C for 40 s, and 72°C for 1 min; subsequently 35 cycles of 94°C for 40 s, 51°C for 40 s, and 72°C for 1 min, with a final extension at 72°C for 5 min. The PCR mix was made in a final volume of 12.5 µl, containing 2 μl of tempered DNA. Successful amplicons were sent for bidirectional sequencing, and BigDye Terminator Cycle Sequencing (v3.1) was used for purification. Sequences were run on an ABI 3730XL DNA sequencer. Forward and reverse sequences were assembled and edited using Codon Code Aligner v 5.0.1. program. The 256 sequences obtained were deposited in the BOLD Systems^®^ database (Ratnasingham & Hebert, 2007) with the following process ID MRRO001‐16 to MRRO285‐16 and MRRO286‐17 to MRRO307‐17.

### 2.4 Sequences analysis

All sequences obtained in this study were compared with sequences available in the BOLD Systems^®^ using “Identification Engine” and with sequences available in GenBank with Basic Local Alignment Search Tool (BLAST) (blast.ncbi.nlm.nih.gov/Blast.cgi). MEGA v 6.06 program (Tamura, Stecher, Peterson, Filipski, & Kumar, 2013) was used for sequences alignment, for searching for stop codons and for the calculation of intra‐ and interspecific genetic distances using the Kimura two parameters distance model (K2P) (Kimura, 1980; Tamura et al., 2013). To provide a graphic representation of the grouping pattern between the different species a K2P distance similarity tree was constructed using the Neighbor‐Joining model (NJ) with Bootstrap support values calculated with 1,000 replicates (Laurito et al., 2013; Rozo‐López & Mengual, 2015; Saitou & Nei, 1987).

### 2.5 Sequences adding from databases

Seventy‐one high‐quality public sequences belonging to 13 species were selected from the BLAST and Identification Engine analysis and added for the construction of the tree; specimens were collected in other geographic sites and identified as some of the species reported in Supporting Information Appendix S1. Only sequences belonging to the BOLD Systems database were added to this study, as this database provides information such as collection data, specimen depository, and identifier. For *Hg. equinus* were adding five sequences generated by us from specimens collected in other locality, theses sequences are still unpublished but have high quality.

### 2.6 Delimitating species

To delimit the Molecular Operational Taxonomic Units (MOTU's), the ABGD (Automatic Barcode Gap Discovery) program was used (Puillandre, Lambert, Brouillet, & Achaz, 2012) with the following parameters: a minimum intraspecific distance (*P*_min_) of 0.001, a maximum intraspecific distance (*P*_max_) that oscillated from 0.02 to 0.1, the barcode gap width parameter with the default configuration (1.5), and the K2P and Jukes–Cantor (JC) (Jukes & Cantor, 1969) evolutionary model. In the present study, an extensive comparison of the partitions resulting from different values of *P*, ranging from 0.010 to 0.045, was performed and a 2.7% threshold value was chosen to delimit each group. The analysis was performed for the sequences obtained for this study and those downloaded from BOLD systems. The BIN system method was also used to delimit the MOTU's in our dataset (Ratnasingham & Hebert, 2013). *Psorophora champerico* sequence was excluded from the ABGD analysis as this is sensitive to singletons.

## 3 RESULTS

### 3.1 Morphological identification

**Figure 2:**
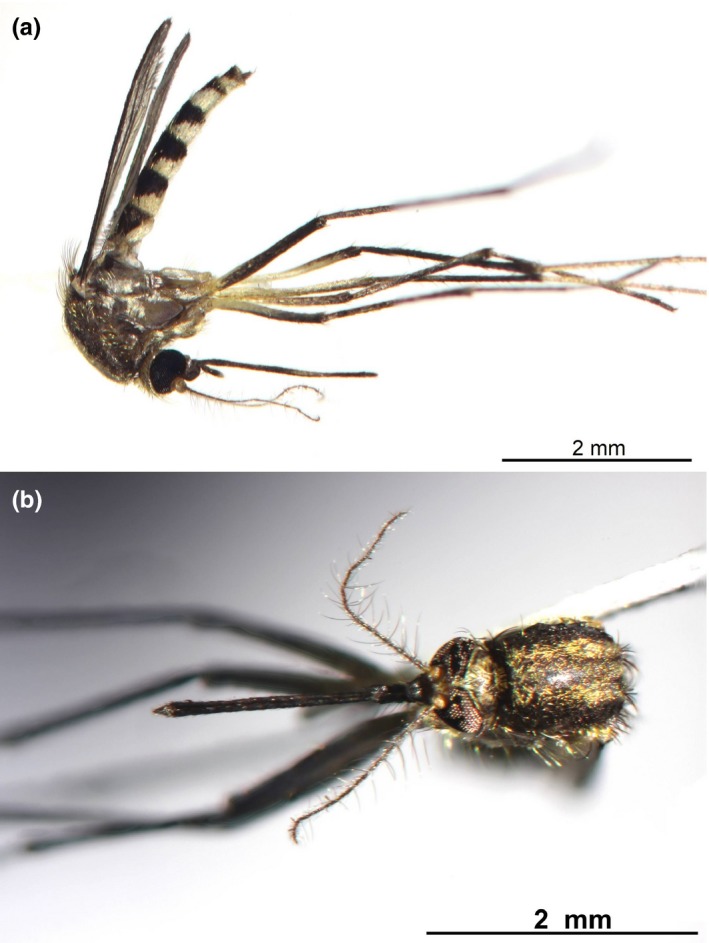
Adult female of *Ae. *n. sp., (a) lateral view (b) dorsal view

A total of 1,413 adult females were collected. Two subfamilies (Anophelinae and Culicinae), four tribes (Aedini, Culicini, Mansoniini, and Sabethini), eight genera (*Anopheles*, *Aedes*, *Haemagogus*, *Psorophora, Culex*, *Coquillettidia*, *Limatus*, and *Wyeomyia*), and 12 subgenera (*Anopheles, Nyssorhynchus, Howardina, Ochlerotatus, Stegomyia, Haemagogus, Janthinosoma, Psorophora, Culex, Melanoconion, Rhynchotaenia,*and *Wyeomyia*). In total, 31 morphospecies were identified (Table 1), within these a new species was discovered *Aedes*n. sp. (Figure 2).

**Table 1:** List of mosquito species, collection sites, and barcode index numbers. Shows average and maximum intraspecific K2P distances observed among COI sequences of mosquito species sequenced in this study. The BINs number by location is Sacxan = 21, Palmar = 24, and Ramonal = 25

Mosquito species	Collection site	BINs	Number of specimens processed	Average K2P distance (%)	Maximum observed K2P distance among conspecific specimens (%)
*Anopheles*(*Anopheles*)* apicimacula* Dyar & Knab, 1906	Site A, Sacxan	BOLD:ACG8818	5	0.10	0.90
	Site A, Palmar	BOLD:ACG8818			
*Anopheles*(*Anopheles*)* crucians*s.l. Wiedemann, 1828	Site A, Sacxan	BOLD:AAC8253	21	1.40	4.40
	Site A, Palmar	BOLD:AAC8253			
	Site A, Ramonal	BOLD:AAC8253			
	Site A, Ramonal	BOLD:ADG0892			
*Anopheles*(*Anopheles*)* pseudopunctipennis* (Theobald, 1901)	Site A, Palmar	BOLD:AAF5940	1	—	—
*Anopheles*(*Anopheles*)* veruslanei* Vargas, 1979	Site A, Palmar	BOLD:ADF9652	5	0.60	1.40
	Site A, Ramonal	BOLD:ADF9652			
*Anopheles*(*Anopheles*)* vestitipennis* Dyar & Knab, 1906	Site A, Palmar	BOLD:AAN4188	13	0.40	1.10
	Site A, Sacxan	BOLD:AAN4188			
	Site A, Ramonal	BOLD:AAN4188			
*Anopheles*(*Nyssorhynchus*)* albimanus* Wiedemann, 1820	Site A, Sacxan	BOLD:AAA3068	31	0.80	2.00
	Site B, Sacxan	BOLD:AAA3068			
	Site A, Palmar	BOLD:AAA3068			
	Site A, Ramonal	BOLD:AAA3068			
*Aedes*(*Howardina*)* cozumelensis* Díaz‐Nájera, 1966	Site A, Ramonal	BOLD:ACO0212	6	0.70	2.10
*Aedes*(*Ochlerotatus*)* angustivittatus* Dyar and Knab, 1907	Site A, Sacxan	BOLD:ACN2972	11	1.00	2.30
	Site A, Palmar	BOLD:ACN2972			
	Site A, Ramonal	BOLD:ACN2972			
	Site B, Ramonal	BOLD:ACN2972			
*Aedes*(*Ochlerotatus*)* euplocamus* Dyar & Knab, 1906	Site A, Palmar	BOLD:ADE4523	11	1.10	2.50
	Site A, Sacxan	BOLD:ADE4523			
	Site A, Ramonal	BOLD:ADE4523			
*Aedes*(*Ochlerotatus*)* fulvus* (Wiedemann, 1828)	Site A, Sacxan	BOLD:ACN9154	2	1.00	1.00
	Site A, Palmar	BOLD:ACN9154			
*Aedes*(*Ochlerotatus*)* scapularis* (Rondani, 1848)	Site A, Sacxan	BOLD:AAH9007	1	—	—
*Aedes*(*Ochlerotatus*)* serratus* (Theobald, 1901)	Site A, Sacxan	BOLD:ACN3711	11	1.80	3.50
	Site A, Palmar	BOLD:ACN3711			
	Site A, Ramonal	BOLD:ACN3711			
*Aedes*(*Ochlerotatus*)* taeniorhynchus* (Wiedemann, 1821)	Site B, Sacxan	BOLD:AAE5975	6	0.80	2.00
	Site A, Palmar	BOLD:AAE5975			
	Site A, Ramonal	BOLD:AAE5975			
*Aedes*(*Ochlerotatus*)* Scapularis Group species*	Site B, Sacxan	BOLD:ACN2972	3	0.50	1.00
	Site A, Ramonal	BOLD:ACN2972			
*Aedes*(*Stegomyia*)* aegypti* (Linnaeus, 1762)	Site A, Sacxan	BOLD:AAA4210	7	0.90	1.90
	Site A, Ramonal	BOLD:AAA4210			
*Haemagogus*(*Haemagogus*)* equinus* Theobald, 1903	Site A, Ramonal	BOLD:ACN9156	7	1.80	3.90
	Site A, Ramonal	BOLD:ADG0616			
	Site A, Ramonal	BOLD:ACN9157			
	Site A, Sacxan	BOLD:ACN9156			
*Psorophora*(*Janthinosoma*)* albipes* (Theobald, 1907)	Site A, Sacxan	BOLD:ACN3418	3	0.70	0.90
	Site B, Sacxan	BOLD:ACN3418			
*Psorophora*(*Janthinosoma*)* cyanescens* (Coquillett, 1902)	Site A, Sacxan	BOLD:AAG3851	11	0.10	1.40
	Site A, Palmar	BOLD:AAG3851			
*Psorophora*(*Janthinosoma*)* champerico* (Dyar & Knab, 1906)	Site B, Sacxan	BOLD:ADE2950	1	—	—
*Psorophora*(*Janthinosoma*)* ferox* (von Humboldt, 1819)	Site A, Sacxan	BOLD:ABZ5766	8	1.50	2.30
	Site A, Palmar	BOLD:ABZ5766			
	Site A, Ramonal	BOLD:ABZ5766			
*Psorophora*(*Janthinosoma*)* lutzii* (Theobald, 1901)	Site A, Sacxan	BOLD:ADE0378	9	0.10	0.90
	Site A, Palmar	BOLD:ADE0378			
	Site A, Ramonal	BOLD:ADE0378			
*Psorophora*(*Psorophora*)* ciliata* (Fabricius, 1794)	Site A, Sacxan	BOLD:AAG3849	7	0.30	1.50
	Site A, Palmar	BOLD:AAG3849			
*Culex*(*Culex*)* coronator*s.l. Dyar & Knab, 1906	Site A, Palmar	BOLD:ADG3686	6	0.30	0.60
	Site A, Ramonal	BOLD:ADG3686			
*Culex*(*Culex*)* interrogator* Dyar & Knab, 1906	Site B, Palmar	BOLD:AAF1735	3	0.00	0.00
	Site A, Ramonal	BOLD:AAF1735			
*Culex*(*Culex*)* nigripalpus* Theobald, 1901	Site A, Sacxan	BOLD:AAF1735	22	0.30	0.70
	Site B, Palmar	BOLD:AAF1735			
	Site A, Ramonal	BOLD:AAF1735			
*Culex*(*Culex*)* quinquefasciatus* Say, 1823	Site A, Sacxan	BOLD:AAA4751	4	0.10	1.00
	Site B, Palmar	BOLD:AAA4751			
*Culex*(*Melanoconion*)* erraticus* (Dyar & Knab, 1906)	Site A, Palmar	BOLD:AAG3848	6	1.80	2.70
	Site A, Ramonal	BOLD:AAG3848			
*Culex*(*Melanoconion*)* taeniopus* Dyar & Knab, 1907	Site A, Palmar	BOLD:AAW1983	7	2.40	3.70
	Site A, Ramonal	BOLD:AAW1983			
*Coquillettidia*(*Rhynchotaenia*)* venezuelensis* (Theobald, 1912)	Site B, Sacxan	BOLD:ADE5089	5	0.50	1.30
	Site A, Palmar	BOLD:ADE5089			
	Site A, Ramonal	BOLD:ADE5089			
*Limatus durhamii* Theobald, 1901	Site B, Sacxan	BOLD:ACN9153	4	0.10	2.40
	Site A, Palmar	BOLD:ACN9153			
	Site B, Palmar	BOLD:ACN9153			
	Site A, Ramonal	BOLD:ACN9153			
*Wyeomyia*(*Wyeomyia*)* celaenocephala* Dyar & Knab, 1906	Site A, Ramonal	BOLD:ACM7671	6	0.00	0.20
	Site A, Palmar	BOLD:ACM7671			

The genus *Aedes* with nine species was the better represented, while *Haemagogus*, *Coquillettidia*, *Limatus*, and *Wyeomyia* were represented by a single species (Table 1). The location Palmar presented the highest number of species (*n* = 25) while Sacxan the smallest (*n* = 22). The most abundant species were *Ae. taeniorhynchus* (*n* = 422), *Ae. serratus*(*n* = 241), and *An. albimanus* (*n* = 125), the three species were present in the three sampled localities, but with greater abundance in Sacxan and Ramonal (Table 2).

**Table 2:** Abundance of species collected by sampling location

Species	Locality			(*n*) Total x spp.
	Sacxan	Palmar	Ramonal	
*Aedes aegypti*	4		5	9
*Aedes angustivittatus*	16	12	23	51
*Aedes cozumelensis*			6	6
*Aedes euplocamus*	48	16	13	77
*Aedes fulvus*	1	1		2
*Aedes scapularis*	1			1
*Aedes serratus*	157	27	57	241
*Aedes*n. sp.	1		2	3
*Aedes taeniorhynchus*	145	125	152	422
*Anopheles albimanus*	46	46	33	125
*Anopheles apicimacula*	1	5	1	7
*Anopheles crucians*s.l.	33	16	27	76
*Anopheles pseudopunctipennis*		1		1
*Anopheles veruslanei*		4	1	5
*Anopheles vestitipennis*	3	6	11	20
*Coquillettidia venezuelensis*		2	3	5
*Culex coronator*s.l.		5	1	6
*Culex erraticus*		4	49	53
*Culex interrogator*		2	1	3
*Culex nigripalpus*	4	50	5	59
*Culex quinquefasciatus*	2	2		4
*Culex taeniopus*		2	13	15
*Haemagogus equinus*	1		20	21
*Limatus durhamii*	6	2	1	9
*Psorophora albipes*	40	9	8	57
*Psorophora champerico*	1			1
*Psorophora ciliata*	4	3		7
*Psorophora cyanescens*	29	20		49
*Psorophora ferox*	25	21	17	63
*Psorophora lutzii*	4	2	3	9
*Wyeomyia celaenocephala*		1	5	6
Total specimens by location	572	384	457	

### 3.2 DNA barcodes

From the 272 specimens processed, 243 COI sequences were obtained. 29 sequences were discarded due to their low quality but the 31 morphologically identified species are represented by their corresponding DNA barcode. The size of the sequences obtained was between 570 and 650 bp. The total number of specimens morphological and molecularly analyzed is reported in Table 1.

The match between the sequences obtained in this study and those deposited in the BOLD Systems^®^ database showed that 25 out of the 31 species presented a 98.02%–100% similarity with conspecific sequences, while in Genbank only 14 species showed a 98%–100% identity (Supporting Information Appendix S2). Sequences obtained in this study for the species *Ae. *n. sp., *Cx. coronator*s.l., *Cx. interrogator*, *Cx. nigripalpus,* and *Cx. quinquefasciatus*, showed ambiguous identification in both databases (Supporting Information Appendix S2). The sequences generated for *An. crucians*s.l., *An. veruslanei*, *An. vestitipennis, Ae. cozumelensis*, *Ae. fulvus*, *Hg. equinus*, *Ps. albipes*, *Ps. lutzii,* *Ps. ciliata*, *Cq. venezuelensis*, and *Wy. celaenocephala* are new to GenBank (Supporting Information Appendix S2). The sequence generated in this study for the species *Ps. champerico* is a new contribution to both databases.

### 3.3 Genetic distance

In this study, the average K2P intraspecific distance was 0.75%. The maximum observed K2P distance between conspecific specimens was for *An. crucians*s.l. with a value of 4.40% (Tables 1 and 3). The maximum interspecific distance for *Ae. serratus* was 3.90% and the same value for *Hg. equinus* (3.90%); for *Ps. ferox* was 4.5% and an average distance of 3.03% was observed between sequences generated here and sequences downloaded from BOLD; for *Cx. taeniopus*a 3.7% distance was observed (Table 1). In *Cx. erraticus* the average distance was 4.1%; for *An. apicimacula* a maximum distance of 8.10% was observed in sequences generated here and sequences downloaded from BOLD; for *Cx. interrogator* and *Cx. nigripalpus,* the average interspecific K2P distance between the specimens of both species was 0.1%; finally, for *Ae. angustivittatus* and *Ae. *n. sp. the average was 1.1% with a maximum interspecific distance of 1.8%.

**Table 3:** K2P sequence divergence of COI barcode region among the mosquito species with >2 specimens, analysis in Culicidae family among the four genera with two or more species

Distance class	*n*	Taxa	Comparisons	Min (%)	Mean (%)	Max (%)
Intraspecific	240	28	1,479	0.00	0.75	4.40
Congeners	221	4	4,945	0.00	7.75	18.00
Confamilial	243	1	22,979	8.74	13.50	22.40

### 3.4 Neighbor‐joining tree

**Figure 3:**
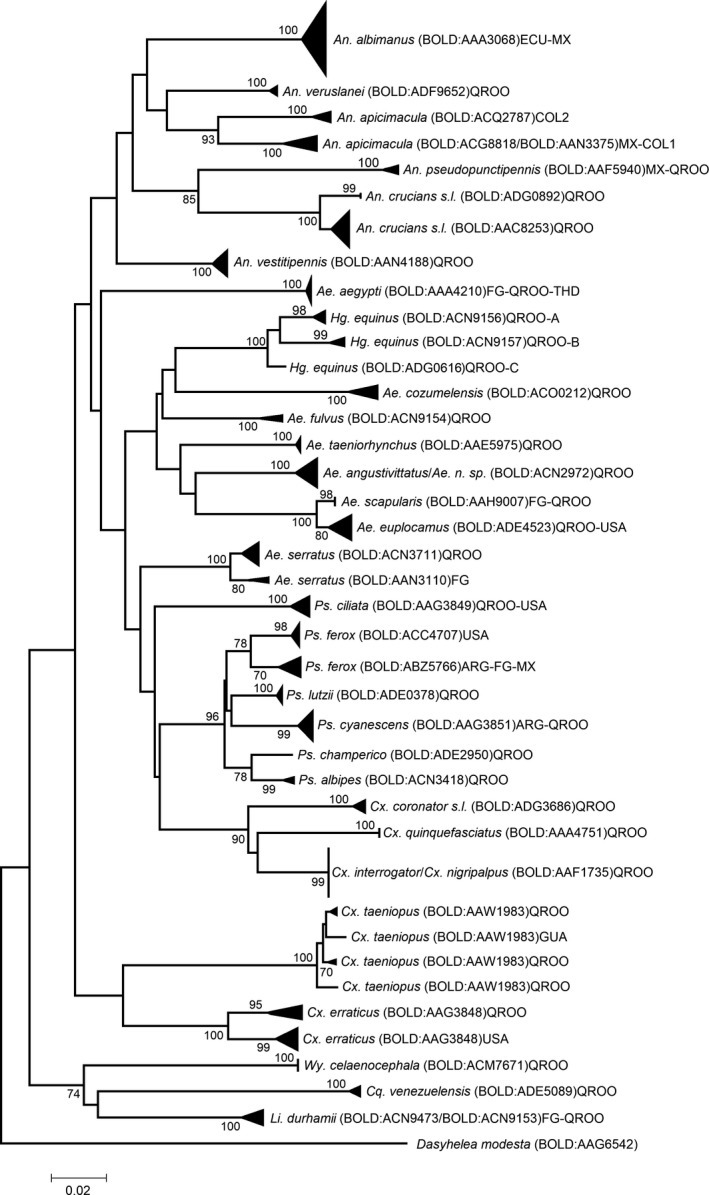
Neighbor‐joining tree based on the Kimura two‐parameter distances among COI sequences (570–650 bp fragment) of 31 Culicidae species. Bootstrap values are shown at the branch points. ARG: Argentina; COL: Colombia; ECU: Ecuador; FG: French Guyana; GUA: Guatemala; MX: mosquito sequences of Quintana Roo and another state of Mexico; QROO: mosquito sequences of this study; THD: Thailand; USA: mosquito sequences of United States of America

Most species formed well‐supported groups in the NJ tree with bootstrap values between 99% and 100%. (Figure 3). The main branches of the NJ tree represented different taxonomic groups such as genera and subgenera. The sequences obtained from BOLD Systems for *An. pseudopunctipennis*, *An. albimanus*, *Ae. euplocamus*, *Ae. scapularis*, *Ae. aegypti*, *Ps. cyanescens*, *Ps. ciliata*, and *Li. durhamii*formed monophyletic groups with the sequences generated in this study, the branches were supported with high bootstrap values (99%–100%) (Figure 3).

Monophyletic clades were observed for *Cx. interrogator* and *Cx. nigripalpus*, and for *Ae. angustivittatus* and *Ae. *n. sp., with high bootstrap values (99%–100%) (Figure 3). *Ae. serratus* formed well‐defined paraphyletic clades (Figure 3) including both the sequences generated in this study (clade QROO) and the sequences from the database belonging to specimens collected by Talaga et al. (2017) in French Guiana (clade FG) (Supporting Information Appendix S1). *Hameagogus equinus* formed a paraphyletic clade, eight sequences (CUL072‐13, CUL079‐13, CUL078‐13, MRRO048‐16, MRRO215‐16, MRRO216‐16, MRRO217‐16, and MRRO221‐16) formed clade QROO‐A supported by a 98% bootstrap value, and other isolated clade (QROO‐B) that included the sequences CUL076‐13, CUL077‐13, and MRRO220‐16, and a third clade (QROO‐C) formed by a single sequence MRRO218‐16. In *Cx. erraticus,* we also found two well‐supported clades, QROO clade with six sequences generated in this study and US clade that include the sequences obtained from the database of specimens collected from Florida (Supporting Information Appendix S1) (Figure 3). For *Ps. ferox* two clades were observed, one consisting of sequences generated in this study mix with sequences downloaded from database and collected in French Guiana, Argentina, and Mexico (Supporting Information Appendix S1) (Figure 3), and a second clade formed by seven sequences belonging to US specimens (Supporting Information Appendix S1); both clades with bootstrap values >60%. Finally, for *An. apicimacula*, were formed two clades, one formed by the sequences generated here and the sequences GBANO930‐14 and GBANO931‐14 from Colombian specimens, and another clade formed by other sequences from Colombian specimens (Supporting Information Appendix S1) (Figure 3).

### 3.5 Delimitation of entities

The ABGD method identified 27 MOTU's for the 31 species (Table 4). *Ae*. *angustivittatus*and *Ae. *n. sp. were assigned to the same MOTU, in the same way for *Ae. euplocamus* and *Ae. scapularis*; *Cx. interrogato*r and *Cx. nigripalpus*; *Ps. albipes* with *Ps. cyanescens*, and *Ps. ferox* with *Ps. lutzii*. On the other hand, the sequences of *An. apicimacula* and *Hg. equinus* were grouped into 2 and 3 different MOTU's, respectively. Derived from the ABGD analysis a distance (K2P) of 2.7% was assigned as a threshold value for assigning each species to its corresponding MOTU, as well as the value for species delimitation.

**Table 4:** MOTUs recovered from COI of Culicidae by ABGD method in this study

Marker	BIN system	Models	ABGD																	
			0.0200^a^		0.0210		0.0220		0.0230		0.0240		0.0250		0.0260		0.0270		0.0280	
			I	R	I	R	I	R	I	R	I	R	I	R	I	R	I	R	I	R
COI	32	K2P	23	27	23	27	23	27	23	27	23	27	23	27	23	27	23	27	23	25
		JC	23	27	23	27	23	27	23	27	23	27	23	27	23	27	23	27	23	25

Note

I: initial partitions; R: recursive partitions.

Number of ABGD partitions obtained by JC and K2P models was the same. Relative gap Width (X) was 1.5.

a Prior intraspecific divergence (*P*).

Unlike 27 MOTUs assigned with ABGD, 32 BINs were assigned by BOLD for the sequences generated in this study (Tables 1 and 2). *Ae. angustivittatus* and *Ae. *n. sp. shared the same BIN (BOLD:ACN2972), as did *Cx. interrogator* and *Cx. nigripalpus* (BOLD:AAF1735) (Table 1). *An. crucians* was divided into two BINs (BOLD:AAC8253 and BOLD:ADG0892) and *Hg. equinus* into three BINs (BOLD:ACN9156, BOLD:ADG0616, and BOLD:ACN9157) (Table 1).

## 4 DISCUSSION

### 4.1 Distribution of anthropofagous species in the border Mexico‐Belize

In the locality of Sacxan, six species of Culicidae had already been reported (Ortega‐Morales et al., 2015), and 17 more species are reported in this study (Table 2), recording a total of 23 species in this locality. Of the six species previously reported, the presence of five of them is confirmed (*An. crucians*s.l., *An. vestitipennis*, *An. albimanus*, *Ps. ferox,* and *Cx. nigripalpus*).

Until now, the diversity of mosquitoes in the areas of Palmar and Ramonal was unknown. The 25 and 24 species reported, respectively, in each area, highlight the importance of biodiversity studies and the correct identification of species that have medical importance (Azari‐Hamidian et al., 2010).

The most important medical species collected in our study in the border zone between Mexico and Belize are *An. pseudopunctipennis*, *An. vestitipennis*, *An. albimanus,* and *Ae. aegypti*. For *An. pseudopunctipennis*, the last report in Quintana Roo dates back more than 60 years (Vargas & Martínez‐Palacios, 1950), and its collection in this study suggests that their distribution in Quintana Roo is wide, this species together with *An. vestitipennis* and *An. albimanus,* are considered the main malaria vector in Mexico (Hiwat & Bretas, 2011; Loyola, Arrendondo, Rodríguez, Browns, & Vasa Marin, 1991; Mijares, Pérez Pacheco, Tomás Martínez, Cantón, & Ambrosio, 1999). By another hand, *Aedes aegypti*is the main vector of Dengue, Chikungunya, and Zika virus in Quintana Roo (Sánchez‐Rodríguez et al., 2014; Torres‐Avendaño et al., 2015). Hence, the importance to continue with the entomological surveillance of these species in the region.

### 4.2 Genetic distance

In the present study, the overlap observed between the intra‐ and interspecific K2P distance in sequences of *Culex* and *Aedes* genera gave rise to ambiguous identifications when using DNA barcoding. Most of the conspecific individuals in those genera showed a genetic distance <2.7%. Species such as *Cx. interrogator* versus *Cx. nigripalpus*, *Ae. angustivittatus* versus *Ae. *n. sp. could not be separated, in both cases we suspect a recent divergence or a high species richness with insufficient sampling.

### 4.3 DNA barcodes and delimitation of species

Twenty‐eight out of 31 species identified morphologically in this study, showed correspondence between morphology and molecular data. Considering all the criteria for delimiting species in this study, we report 33 taxonomic entities (31 morphospecies, 1 cryptic species in *Hg. equinus,* and 1 cryptic species in *An. crucians*s.l.). For the single sequence of *Hg. equinus* that form an aisled clade, is necessary to collect specimens and add sequences, but we do not discart the possibility of existence of more cryptic specie.

For *Ae. euplocamus* and *Ae. scapularis;* *Ps. albipes* and *Ps. cyanescens;* *Ps. ferox* and *Ps. lutzii* grouped in the same MOTU by the ABGD method, but assigned in a different BIN number for each species by BOLD systems. The discordance between the two MOTUs delimitation methods, might be due to the different threshold values used for separating species, 2.2% by default for the BIN system method, while 2.7% was considered for the ABGD (see Section 2.6). Although has been pointed that the taxonomic performance of RESL in BIN system is stronger that ABGD algorithm, showing better results between species identified and MOTU assigned (Ratnasingham & Hebert, 2013). In ABGD, the results improve when is selected a best partitioning scheme, in the analysis the final partitioning depends to a large extent on the user‐defined prior upper limit to intraspecific distance (*P*) as pointed out by Puillandre et al. (2012). Also, it is important to mention that both analyses largely depend on the representation of the species (Pentinsaari, Vos, & Mutanen, 2017).

The results obtained with the ABGD method in this study are similar to those obtained by Versteirt et al. (2015) for the culicids of Belgium, where *Cx. pipiens* and *Cx. torrentium, Ae. cantans* and *Ae. annulipes, Ae. punctor* and *Ae. communis*, and *An. maculipennis* s.s. and An. messeae were grouped in the same MOTU. In Aedes genera, the intraspecific divergence was between 3.6% and 3.9%, evidencing that the delimitation of species in this genera depends largely on the sampling (Beebe, 2018).

### 4.4 Cryptic diversity

#### 4.4.1 *Anopheles apicimacula*

Our results supported the hypothesis of Gómez, Bickersmith, González, Conn, and Correa (2015), suggesting that *An. apicimacula* is a species complex. In this study, when analyzing the sequences generated here and those of specimens from Colombia, the average intraspecific distance (K2P) was 5.6%. Our sequences formed a clade with sequences of *An. apicimacula* from Antioquia Colombia, while sequences of *An. apicimacula* from Choco and Valle del Cauca Colombia formed another clade. Due to the proximity between our study area (<300 km) and the type locality (Livingston, Guatemala), it is likely that our collected specimens corresponded to *An. apicimacula*s.s. (Dyar & Knab, 1906) (Colombian Caribbean lineage), while the specimens that form the lineage of the Colombian Pacific (Choco and Valle del Cauca) belong to the new species (*An. apicimacula*s.l.).

#### 4.4.2 *Anopheles crucians* s.l.

In Mexico, two species of this complex have been reported using only morphology, *An. crucians* s.l. which is reported in North America (Massachusetts to New Mexico, USA), Central America (Mexico to Nicaragua) and in the Caribbean islands (Wilkerson, Reinert, & Li, 2004), and An. bradleyi which is distributed along the coasts of the Atlantic and Gulf of the USA up to the south of Nicaragua (Floore, Harrison, & Eldridge, 1976). It is necessary to continue studying *An. crucians* species and contribute with information in order to facilitate the separation of all the species of the complex. The genetic distances of 4.4% reported among the specimens collected in this study, suggested the presence of cryptic speciation. This result was not surprising as *An. crucians* has been previously considered as a complex formed by seven species which are impossible to separate using only adult female characters, and therefore, the use of molecular markers could represent the only way to allow their separation (Wilkerson et al., 2004). However, with the results obtained in this study, we might probably report an eighth species in this complex.

#### 4.4.3 *Aedes serratus*

This species showed a greater intraspecific genetic distance than the one previously reported (3.50%); furthermore, the distance between specimens collected in this study (*Ae. serratus* QROO) and sequences of individuals collected in French Guiana (*Ae. serratus* FG) showed the existence of two lineages. So far, a wide distribution has been reported for this species from Mexico to Brazil (WRBU, 2018) but it is very likely that this wide distribution will be interrupted by the Andes mountain range that crosses Colombia and Venezuela which is responsible for propitiating the speciation (De‐Silva et al., 2017).

#### 4.4.4 *Hameagogus equinus*

The three clades observed with an average intraspecific distance of 3.05% also suggest cryptic speciation within the genus. No morphological variation has yet been found in the analyzed specimens but we suggest including the observation of immature stages such as eggs, larvae, and pupae, as well as the genitalia of the adult males to find the trait(s) that separate these species (Versteirt et al., 2015). Five specimens of *Hg. equinus* included in this analysis were collected in 2013 in a locality separated by 30 km of actual study area, four on five specimens were grouped with QROO‐A clade and one with QROO‐B clade, support our hypothesis that there are more than one species within *Hg. equinus*.

#### 4.4.5 *Psorophora ferox*

An average K2P distance of 3.23% was observed for *Ps. ferox,* which separates specimens from Florida from those from Mexico, Argentina, and French Guiana, forming two well‐defined clades. Recently, Mello, Santos‐Mallet, Tátila‐Ferreira, and Alencar (2018) found significant differences in the external morphology of the eggs, the exochorion ornamentation, in three populations (the USA, Trinidad and Tobago, and Brazil). These differences were pointed out by Linley and Chadee (1990) in the population of Southeast USA and Trinidad and Tobago; however, they were not considered by the separation of species. Nevertheless, we now show molecular evidences that suggest more than one lineage for *Ps. ferox*. These results imply the necessity to continue with studies including integrative taxonomy of *Ps. ferox* populations throughout the American continent, which would help delimiting new species and establishing their geographic range (Dayrat, 2005; Will, Mishler, & Wheeler, 2005).

#### 4.4.6 *Culex erraticus*

Mendenhall, Bahl, Blum, and Wesson (2012) identified two main lineages in *Cx. erraticus* from USA and other countries of America, by analyzing sequences of the Internal Transcribed Spacer 2 (ITS2) and the mitochondrial genes from NADH dehydrogenase. The analysis showed that a lineage represents Central and Eastern USA, while the other corresponds to Central America, South America, and West USA. In this study, the generated sequences are grouped in one clade and the sequences of specimens from Florida (USA) in another clade with an average K2P distance of 4.1%. This result showed a strong evidence of the existence of two lineages for *Cx. erraticus*. By this way, our results reinforce the hypothesis of Mendenhall et al. (2012) that there are physical barriers such as the Chihuahuan desert that limit the dispersal of *Cx. erraticus* and is reasonable to think that larvae develop is limited to bodies of semipermanent and permanent waters that include ditches, flood areas, streams (WRBU, 2018).

#### 4.4.7 *Culex taeniopus*

The usefulness of DNA barcodes for the identification of species of the subgenus *Melanoconion* has been reported, as well as the discovery of cryptic species or new species within the genus *Culex* (Laurito et al., 2013; Torres‐Gutiérrez et al., 2016). Values of 3.70% are reported for specimens of *Cx. taeniopus* collected for this study. The specimens were collected in relatively close localities (Palmar and Ramonal), with a distance between localities of 12 km, and no apparent geographic barrier that can lead to speciation was present; therefore, the cryptic speciation could be the result of a sympatric speciation. To clarify the high values of intraspecific divergence, we suggest extend the collection of specimens to increase the number of sequences and represent a greater geographical area, at this moment Guatemala is only represented with one specimen, performing ecological studies and observation of structures such as genitalia (in males) and the morphology of larvae and eggs to find any character that could separate the species. It is in our interest to rule out the possibility of the presence of a complex of species and not only cryptic speciation.

### 4.5 Morphological differences versus low genetic distances

The interspecific distance between *Ae. angustivittatus*and *Ae. *n. sp. of 1.1%, the low average is due that probably both species belong to the Scapularis group. However, morphological characters of *Ae. *n. sp. did not match those of *Ae. angustivittatus,*nor those of *Ae. trivittatus*or the characters of the hybrid reported by Arnell (1976) originating from *Ae. angustivittatus* and *Ae. trivittatus. Aedes angustivittatus*and *Ae. *n. sp. species presented differences in the pattern of ornamentation of the scutum. Females of *Ae. angustuvuttatus* presented two longitudinal lines of pale golden scales along the scutum (Arnell, 1976; Clark‐Gil & Darsie, 1983). *Aedes*n. sp. presented three wide lines of golden scales, one covering the acrosthical line and the other two placed each on one side of the acrosthical line, similar to those reported by Arnell (1976) for *Ae. crinifer*. The distribution of *Ae. crinifer* is restricted to South America (Arnell, 1976; WRBU, 2018), and sequences of *Ae. crinifer* from Argentina obtained by Díaz‐Nieto et al., (2013) were analyzed and the average intraspecific distance between *Ae. crinifer* and our *Ae. *n. sp. was 8.3%.

Another case of incomplete separation is *Cx. interrogator* and *Cx. nigripalpus*, COI gene did not present enough divergence to separate the two species, as the distance between both species was 0.1%. Though similar cases were observed within species of *Culex* in South America, where 22 species were sequenced but 30% could not be delimited despite using multilocus (COI or ITS2) (Laurito et al., 2013). The same case was reported between *Culex minor* and *Culex spiculosus*, which presented an average K2P interspecific distance of 1.86% (Wang et al., 2012). These results could be explained by an incomplete lineage sorting or introgression events (Beebe, 2018).

Cases like those here reported, where COI does not present enough divergence to separate close species because the lineages are not fully sorted into divergent clades, are not new, but our data increase the lineages record of recent divergence. In this sense, it is important to conduct further studies in order to look for evidence of reproductive isolation. Also, the use of a single gene region is an imperfect tool because will be overlooked recent species because of their low sequence divergence (Ratnasingham & Hebert, 2013). It is necessary to include at the analysis nuclear marker like ITS2 as well as ecological and biological data (Ajamma et al., 2016; Alquezar, Hemmerter, Cooper, & Beebe, 2010; Beebe, 2018; Mardulyn, Othmezouri, Mikhailov, & Pasteels, 2011; Versteirt et al., 2015; Wang et al., 2012).

## 5 CONCLUSIONS

The results in this study contribute to the development of a reference DNA barcode library for Mexican culicids. In addition, our analysis reported the presence of a certain taxonomic differentiation that needs to be investigated in *An. apicimacula*, *An. crucians*s.l.*,* *Ae. serratus*, *Hg. equinus*, *Ps. ferox*, *Cx. erraticus*, and *Cx. taeniopus*. For the new specie discovered, it is necessary to work on the description during all their life stages (larval exuvia, pupa, and adults), which allow the documentation of the link between morphological and molecular identification standards (Versteirt et al., 2015). Finally, the identification of species is an essential step for monitoring and vectors control. This information is valuable for the Ministry of Health (SS) of Mexico for the contribution in the epidemiological surveillance and the design of programs for the control of vector‐borne diseases in the border region with Belize.

## CONFLICT OF INTEREST

None declared.

## AUTHOR CONTRIBUTIONS

R.J.C.‐C. involved in samplings, taxonomic identification, and analysis of genetic distances. A.M.‐A. generated the sequence data and its genetic analysis. A.M.‐A. P.C.M.‐A. designed the study. A.I.O.‐M. provided taxonomic support on identification. All authors contributed to the text and approved the final manuscript.

## Data Availability

The collected data are deposited in the database BOLD Systems^®^ (www.boldsystems.org) in the project “Mosquitoes from Rio Hondo, Mexican Caribbean” (MRRO). Molecular sequences have been submitted to GenBank on June 13.
