# Supplementary material for: DNA barcodes and evidence of cryptic diversity of anthropophagous mosquitoes in Quintana Roo, Mexico
Source: Ecol Evol. 2019 Mar 23;9(8):4692–705. doi: 10.1002/ece3.5073 (PMC6476762; doi:10.1002/ece3.5073)
Supplement: Supplementary file 1 [file ECE3-9-4692-s001.docx]

**Appendix 1**. Sequences downloaded from BOLD of 14 species that were added to the analysis in this study. * = no-public sequences generated by us from another study.

| Species | (n) sequences | Process ID | BIN | Country | State/collection site |
| --- | --- | --- | --- | --- | --- |
| *Ae. aegypti* | 4 | FGMOS1063-16 | BOLD:AAA4210 | French Guiana | Matoury |
|  |  | GBDCU1627-15 | BOLD:AAA4210 | Thailand | ----- |
|  |  | GBDCU1628-15 | BOLD:AAA4210 | Thailand | ----- |
|  |  | GBDCU1629-15 | BOLD:AAA4210 | Thailand | ----- |
| *Ae. euplocamus* | 1 | BBDIT1101-11 | BOLD:ADE4523 | USA | Texas |
| *Ae. scapularis* | 3 | FGMOS923-16 | BOLD:AAH9007 | French Guiana | Kourou |
|  |  | FGMOS925-16 | BOLD:AAH9007 | French Guiana | Kourou |
|  |  | FGMOS1173-16 | BOLD:AAH9007 | French Guiana | Kourou |
| *Ae. serratus* | 3 | FGMOS931-16 | BOLD:AAN3110 | French Guiana | Kourou |
|  |  | FGMOS933-16 | BOLD:AAN3110 | French Guiana | Kourou |
|  |  | FGMOS1171-16 | BOLD:AAN3110 | French Guiana | Kourou |
| *An. albimanus* | 6 | GBMIN14511-13 | BOLD:AAA3068 | Ecuador | ------ |
|  |  | GBMIN14517-13 | BOLD:AAA3068 | Ecuador | ------ |
|  |  | GBMIN14518-13 | BOLD:AAA3068 | Ecuador | ------ |
|  |  | MEXSM065-12 | BOLD:AAA3068 | Mexico | Chiapas |
|  |  | MEXSM071-12 | BOLD:AAA3068 | Mexico | Chiapas |
|  |  | MEXSM072-12 | BOLD:AAA3068 | Mexico | Chiapas |
| *An. apicimacula* | 7 | GBANO893-14 | BOLD:ACQ2787 | Colombia | Valle del Cauca |
|  |  | GBANO894-14 | BOLD:ACQ2787 | Colombia | Valle del Cauca |
|  |  | **GBANO910-14** | BOLD:ACQ2787 | Colombia | Choco |
|  |  | **GBANO911-14** | BOLD:ACQ2787 | Colombia | Choco |
|  |  | GBANO912-14 | BOLD:ACQ2787 | Colombia | Choco |
|  |  | GBANO930-14 | BOLD:AAN3375 | Colombia | Antioquia |
|  |  | GBANO931-14 | BOLD:AAN3375 | Colombia | Antioquia |
| *An. pseudopunctipennis* | 3 | MEXSM077-12 | BOLD:AAF5940 | Mexico | Chiapas |
|  |  | MEXSM079-12 | BOLD:AAF5940 | Mexico | Chiapas |
|  |  | MEXSM082-12 | BOLD:AAF5940 | Mexico | Chiapas |
| *Cx. erraticus* | 11 | IUP966-14 | BOLD:AAG3848 | USA | Florida |
|  |  | IUP967-14 | BOLD:AAG3848 | USA | Florida |
|  |  | IUP968-14 | BOLD:AAG3848 | USA | Florida |
|  |  | IUP969-14 | BOLD:AAG3848 | USA | Florida |
|  |  | IUP970-14 | BOLD:AAG3848 | USA | Florida |
|  |  | NEONU1502-12 | BOLD:AAG3848 | USA | Florida |
|  |  | NEONU1503-12 | BOLD:AAG3848 | USA | Florida |
|  |  | NEONU1576-12 | BOLD:AAG3848 | USA | Florida |
|  |  | NEONU1577-12 | BOLD:AAG3848 | USA | Florida |
|  |  | NEONU1601-12 | BOLD:AAG3848 | USA | Florida |
|  |  | NEONU1607-12 | BOLD:AAG3848 | USA | Florida |
| *Cx. taeniopus* | 1 | NEONV050-11 | BOLD:AAW1983 | Guatemala | ------- |
| *Hg. equinus** | 5 | CUL072-13 | BOLD:ACN9156 | Mexico | Quintana Roo |
|  |  | CUL076-13 | BOLD:ACN9157 | Mexico | Quintana Roo |
|  |  | CUL077-13 | BOLD:ACN9157 | Mexico | Quintana Roo |
|  |  | CUL078-13 | BOLD:ACN9156 | Mexico | Quintana Roo |
|  |  | CUL079-13 | BOLD:ACN9156 | Mexico | Quintana Roo |
| *Li. durhamii* | 3 | FGMOS133-16 | BOLD:ACN9473 | French Guiana | Kourou |
|  |  | FGMOS643-16 | BOLD:ACN9473 | French Guiana | Kourou |
|  |  | FGMOS644-16 | BOLD:ACN9473 | French Guiana | Kourou |
| *Ps. ciliata* | 3 | IUP935-14 | BOLD:AAG3849 | USA | Florida |
|  |  | IUP937-14 | BOLD:AAG3849 | USA | Florida |
|  |  | IUP938-14 | BOLD:AAG3849 | USA | Florida |
| *Ps. cyanescens* | 5 | CBMA085-12 | BOLD:AAG3851 | Argentina | ------- |
|  |  | CBMA141-12 | BOLD:AAG3851 | Argentina | ------- |
|  |  | EFLP158-12 | BOLD:AAG3851 | Argentina | ------- |
|  |  | EFLP159-12 | BOLD:AAG3851 | Argentina | ------- |
|  |  | GMAFE073-15 | BOLD:AAG3851 | Argentina | ------- |
| *Ps. ferox* | 21 | GBDP8484-10 | BOLD:AAO0580 | Argentina | ------- |
|  |  | GBDP8485-10) | BOLD:AAO0580 | Argentina | ------- |
|  |  | FGMOS1178-16 | BOLD:AAO0580 | French Guiana | Kourou |
|  |  | FGMOS926-16 | BOLD:AAO0580 | French Guiana | Kourou |
|  |  | FGMOS934-16 | BOLD:AAO0580 | French Guiana | Kourou |
|  |  | MEXAO016-14 | BOLD:ABZ5766 | Mexico | San Luis Potosi |
|  |  | IUP831-14 | BOLD:ACC4707 | USA | Massachusetts |
|  |  | IUP836-14 | BOLD:ACC4707 | USA | Massachusetts |
|  |  | IUP837-14 | BOLD:ACC4707 | USA | Massachusetts |
|  |  | IUP838-14 | BOLD:ACC4707 | USA | Massachusetts |
|  |  | IUP944-14 | BOLD:ACC4707 | USA | Florida |
|  |  | IUP945-14 | BOLD:ACC4707 | USA | Florida |
|  |  | IUP946-14 | BOLD:ACC4707 | USA | Florida |
|  |  | IUP947-14 | BOLD:ACC4707 | USA | Florida |
|  |  | IUP948-14 | BOLD:ACC4707 | USA | Florida |
|  |  | NEONU1517-12 | BOLD:ACC4707 | USA | Florida |
|  |  | NEONU1518-12 | BOLD:ACC4707 | USA | Georgia |
|  |  | NEONU1520-12 | BOLD:ACC4707 | USA | Georgia |
|  |  | NEONU1584-12 | BOLD:ACC4707 | USA | Florida |
|  |  | NEONU1599-12 | BOLD:ACC4707 | USA | Florida |
|  |  | NEONU1606-12 | BOLD:ACC4707 | USA | Florida |
