# Supplementary material for: DNA barcodes and evidence of cryptic diversity of anthropophagous mosquitoes in Quintana Roo, Mexico
Source: Ecol Evol. 2019 Mar 23;9(8):4692–705. doi: 10.1002/ece3.5073 (PMC6476762; doi:10.1002/ece3.5073)
Supplement: Supplementary file 2 [file ECE3-9-4692-s002.docx]

**Appendix 2.** Sequences identification by comparison whith molecular Database Genbank and BOLDSystems. The abbreviation Nw-seq = a new sequence for the database.

| Sequences of species morphologically identified | BOLD Similarity % | Genbank, Identity % |
| --- | --- | --- |
| *Ae. aegypti* | 100.00 *Ae. aegypti* | 100.00 *Ae. aegypti* |
| *Ae. angustivittatus* | 98.84 *Ae. angustivittatus* | 98.00 *Ae. angustivittatus* |
| *Ae. cozumelensis* | 99.80 *Ae. cozumelensis* | Nw-seq |
| *Ae. euplocamus* | 98.87 *Ae. euplocamus* | 98.00 *Ae. euplocamus* |
| *Ae. fulvus* | 99.29 *Ae. fulvus* | Nw-seq |
| *Ae. scapularis* | 100.00 *Ae. scapularis* | 100.00 *Ae. scapularis* |
| *Ae. serratus* | 99.65 *Ae. serratus* | 98.00 *Ae. serratus* |
| *Ae. n. sp.* | 98.84 *Ae. angustivittatus* | 96.00 *Ae. angustivittatus* |
| *Ae. taeniorhynchus* | 99.82 *Ae. taeniorhynchus* | 99.00 *Ae. taeniorhynchus* |
| *An. albimanus* | 99.51 *An. albimanus* | 99.00 *An. albimanus* |
| *An. apicimacula* | 99.84 *An. apicimacula* | 98.00 *An. apicimacula* |
| *An. crucians s.l.* | 98.02 *An. crucians* | Nw-seq |
| *An. pseudopunctipennis* | 100.00 *An. pseudopunctipennis* | 99.00 *An. pseudopunctipennis* |
| *An. veruslanei* | 99.40 *An. veruslanei* | Nw-seq |
| *An. vestitipennis* | 100.00 *An. vestitipennis* | Nw-seq |
| *Cq. venezuelensis* | 100.00 *Cq. venezuelensis* | Nw-seq |
| *Cx. coronator* *s.l.* | 100.00 *Cx. coronator, Cx. maxi.*  98.80 *Cx. usquatus, Cx. camposi* | 99.00 *Cx. coronator, Cx. usquatus, Cx. maxi* |
| *Cx. erraticus* | 99.68 *Cx. erraticus* | 98.00 *Cx. erraticus* |
| *Cx. interrogator* | 99.00 *Cx. nigripalpus, Cx. interrogator* | 99.00 *Cx. nigripalpus,*  *Cx. mollis, Cx. declarator* |
| *Cx. nigripalpus* | 99.00 *Cx. nigripalpus, Cx. conspirator, Cx. interrogator* | 99.00 *Cx. nigripalpus, Cx. mollis, Cx. cospirator, Cx. declarator* |
| *Cx. quinquefasciatus* | 100.00 *Cx. quinquefasciatus,*  *Cx. pipiens pipiens,* | 99.00 *Cx. quinquefasciatus,*  *Cx. pipiens, Cx. pipiens pallens,*  *Cx. pipiens pipiens* |
| *Cx. taeniopus* | 100.00 *Cx. taeniopus* | 98.00 *Cx. taeniopus* |
| *Hg. equinus* | 99.83 *Hg. equinus* | Nw-seq |
| *Li. durhamii* | 99.80 *Li. durhamii* | 98.00 *Li. durhamii* |
| *Ps. albipes* | 99.49 *Ps. albipes* | Nw-seq |
| *Ps. champerico* | Nw-seq | Nw-seq |
| *Ps. ciliata* | 98.98 *Ps. ciliata* | Nw-seq |
| *Ps. cyanescens* | 99.93 *Ps. cyanescens* | 100 *Ps. cyanescens* |
| *Ps. ferox* | 100.00 *Ps. ferox* | 98.00 *Ps. ferox* |
| *Ps. lutzii* | 99.66 *Ps. lutzii* | Nw-seq |
| *Wy. celaenocephala* | 99.50 *Wy. celaenocephala* | Nw-seq |
